# Non-symbolic estimation of big and small ratios with accurate and noisy feedback

**DOI:** 10.3758/s13414-024-02914-6

**Published:** 2024-07-11

**Authors:** Nicola J. Morton, Matt Grice, Simon Kemp, Randolph C. Grace

**Affiliations:** School of Psychology, Speech and Hearing, University of Canterbury, Christchurch, New Zealand

**Keywords:** Math modelling, Perceptual learning, Similarity

## Abstract

The ratio of two magnitudes can take one of two values depending on the order they are operated on: a ‘big’ ratio of the larger to smaller magnitude, or a ‘small’ ratio of the smaller to larger. Although big and small ratio scales have different metric properties and carry divergent predictions for perceptual comparison tasks, no psychophysical studies have directly compared them. Two experiments are reported in which subjects implicitly learned to compare pairs of brightnesses and line lengths by non-symbolic feedback based on the scaled big ratio, small ratio or difference of the magnitudes presented. Results of Experiment 1 showed all three operations were learned quickly and estimated with a high degree of accuracy that did not significantly differ across groups or between intensive and extensive modalities, though regressions on individual data suggested an overall predisposition towards differences. Experiment 2 tested whether subjects learned to estimate the operation trained or to associate stimulus pairs with correct responses. For each operation, Gaussian noise was added to the feedback that was constant for repetitions of each pair. For all subjects, coefficients for the added noise component were negative when entered in a regression model alongside the trained differences or ratios, and were statistically significant in 80% of individual cases. Thus, subjects learned to estimate the comparative operations and effectively ignored or suppressed the added noise. These results suggest the perceptual system is highly flexible in its capacity for non-symbolic computation, which may reflect a deeper connection between perceptual structure and mathematics.

## Introduction

How we compare stimulus magnitudes that vary along a continuous perceptual dimension – such as brightness, length, loudness or weight – has been an enduring question for psychophysics and experimental psychology. Theoretical accounts have typically assumed that subjects are able to judge either differences or ratios of perceived magnitudes (Birnbaum, 1978, 1978, 1980; Colonius & Dzhafarov, 2006; Dzhafarov & Colonius, 1999, 2005; Luce, 2002; Stevens, 1961, 1975), fostering a debate that has persisted since the earliest theorising about psychophysics (Fechner et al., 1966; Plateau, 1872; Murray, 1993; Scheerer, 1987). However, progress has been hindered because ratios and differences can be transformed into one another by simple mathematical functions (i.e., logarithmic, exponential), so alternative models can be difficult, if not impossible, to distinguish empirically (Torgerson, 1961). A similar mathematical indeterminacy has contributed to the Fechner-Stevens controversy about the psychophysical law (Krueger, 1989; Shepard, 1966). A body of research dating back to the 1970s has attempted to settle the matter using variations on Stevens’ methods of magnitude estimation (Stevens, 1957), category-rating tasks, and non-metric scaling analyses designed to empirically disentangle judged ratios from differences. Across a range of perceptual stimuli, subjects have been instructed to estimate differences and/or ratios of magnitude pairs using either verbal numeric responses or by assigning a rating along an ordinal scale. They have compared heaviness (Birnbaum & Veit, 1974; Mellers et al., 1984; Masin & Brancaccio, 2017; Masin et al., 2019; Rule et al., 1981), brightness (Masin, 2013, 2014; Masin et al., 2019), darkness (Birnbaum, 1978; Veit, 1978), line length (Masin et al., 2019; Parker et al., 1975), loudness (Birnbaum & Elmasian, 1977; Schneider et al., 1976), distances between US cities (Birnbaum & Mellers, 1978), pitch (Elmasian & Birnbaum, 1984), and sweetness (de Graaf & Frijters, 1988). No clear consensus emerged from these studies, although the majority found evidence in favour of a single comparative operation, most commonly differences. More recently, Masin et al. (2019) have proposed that whereas differences between stimulus magnitudes can always be computed, ratio judgements are only possible with respect to extensive dimensions (e.g., line length), and not intensive modalities (e.g., brightness, loudness, heaviness).

A limitation of all prior studies of perceptual comparison, however, and the primary focus of the current paper, is the way in which ratio judgements have been conceptualised. Among the infinitely many possible functions of two positive magnitudes, differences and ratios are obvious candidates for the operation(s) that describe how perceptual comparisons are made. Computationally, their counterparts of addition and multiplication comprise the fundamental operations of arithmetic, and psychologically, these appear to be embedded in basic cognitive processes that are present from infancy and shared across species (Christodoulou et al., 2017; Emmerton, 2001; Hauser et al., 1996; Honig & Stewart, 1989; Howard et al., 2019; McCrink & Wynn, 2007; Rugani et al., 2009; Vallentin & Nieder, 2008). However, when we consider more closely the structural properties of differences and ratios as sets, we find that there are not two possible comparative operations, but three.

**Fig. 1:**
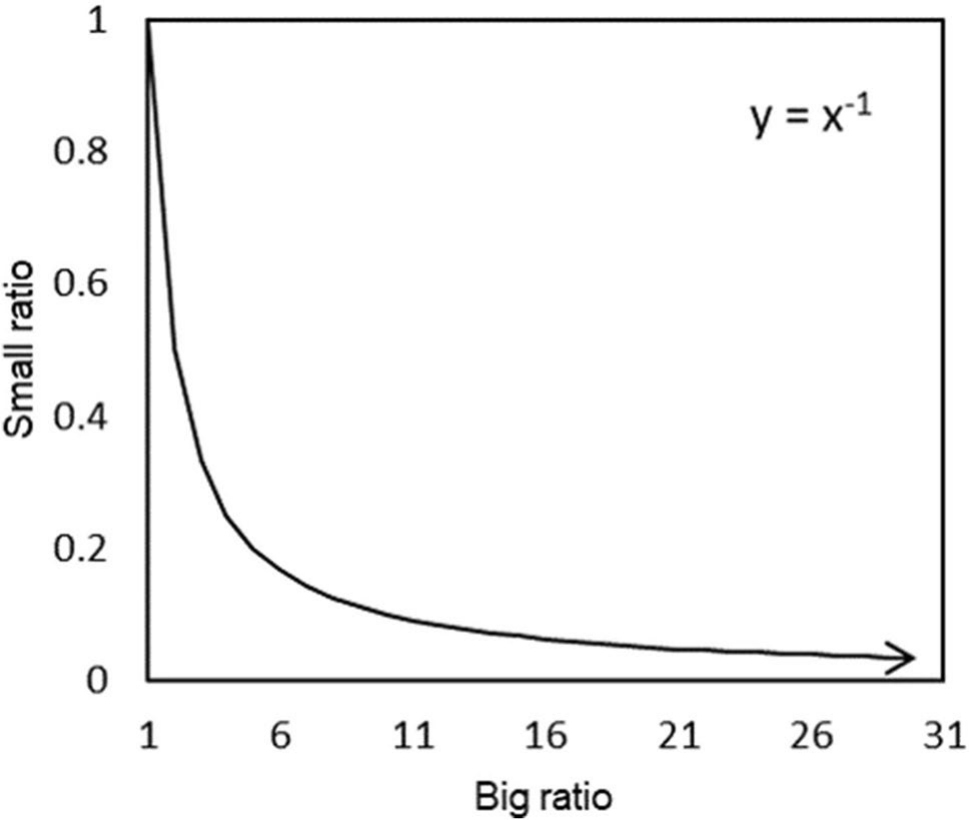
Relationship between big and small ratios, being a subset of the function *y* = *x*^--1^

We can describe the relevant set theoretic properties of differences and ratios by considering all possible outcomes of applying each operation to two positive magnitudes. For differences, the possible outputs range between 0 (where both input magnitudes are equal) and infinity (where the magnitude of one input is arbitrarily large with respect to the other). Although the sign associated with a difference can be positive or negative (depending on the order of the operands), the magnitude of the resulting difference is the same, as is the possible range. In this sense, we can say that differences (as a set) are bounded below by 0 (since their value cannot, in an absolute sense, be less than 0), and unbounded above (since they can potentially take on any positive magnitude). For ratios, the task is less straightforward. Depending again on the order of the operands, there are two ways a ratio of two magnitudes can be quantified. Unlike differences, these give outputs that differ in both magnitude and range. On the one hand, we can take the ratio of the larger magnitude to the smaller magnitude (*M*/*m*, where *m* ≤ *M*), which results in a set with analogous properties to that of differences, bounded below this time by 1 (where *m* = *M*) and unbounded above (where *M* is arbitrarily large with respect to *m*). Let us call this type of ratio a *big ratio*. On the other hand, taking the ratio of the smaller magnitude to the larger magnitude (*m*/*M*, where *m* ≤ *M*) gives a different result. When we consider the set of all possible outputs of this, what we call *small ratio* function, we find that it is bounded below by 0 (where *m* is arbitrarily small with respect to *M*), and above by 1 (where *m* = *M*). A small ratio must always take a value in this range, no matter how divergent its two inputs may be. The set of big ratios, conversely, is unbounded above, and so can take any value greater than 1. The relationship between big ratios and small ratios is therefore non-linear (being a subset of the function *y* = *x*^−1^; see Fig. 1). As the equation describing this relationship indicates, big ratios and small ratios are multiplicative inverses of each other. For example, where *m* = 1 and *M* = 4, the big ratio of the magnitude pair = 4 (4/1), and the small ratio = 0.25 (1/4). Multiplying the big and small ratio of any magnitude pair (in this example 4 and 0.25) always gives 1. In a meaningful sense then, big and small ratios, when applied to a given pair of magnitudes, are simply two ways of expressing the same relationship. As Fig. 1 shows, however, the respective *sets* of big and small ratios have demonstrably different structural properties (specifically, with respect to the Euclidean metric, one set is unbounded above and the other is not). Because perceptual comparison experiments involve sets of, rather than single comparative judgements, these structural differences carry divergent empirical predictions.

To the best of our knowledge, no psychophysical studies to date have explicitly tested the capacity for small ratio estimation of magnitude pairs in isolation. Chesney and Matthews (2018) had participants estimate numerosities of dot clouds by placing a mark on a number line whose ends were marked by a single dot on the left, and a more numerous dot cloud on the right. Chesney and Matthews (2022) ran the same task using circle areas, with the ends of the number line flanked by smaller and bigger circles. Although these were magnitude estimation tasks, rather than magnitude comparison tasks, they effectively required participants to make non-symbolic judgements of the ratio of the magnitude presented to the larger magnitude comprising the right label (i.e., a small ratio judgement).

The psychophysical comparison tasks described by de Graaf and Frijters (1988) and Masin et al. (Masin, 2013, 2014; Masin et al., 2019; Masin & Brancaccio, 2017) employed big ratio judgements only. For example: "[subjects] had to first identify which stimulus of each pair was the sweetest, and subsequently to assign a number reflecting the "ratio" of the perceived sweetness intensity of the sweetest stimulus to the intensity of the least sweet stimulus" (de Graaf & Frijters, 1988, p. 358); and "participants were asked to verbally judge on each trial how many times the variable stimulus was heavier than the standard stimulus" (Masin, 2014, p. 139).

In the other psychophysical comparison studies cited, big and small ratios were usually combined into a single scale, with the order in which the stimulus pairs were presented to subjects dictating whether a big or small ratio should be employed. For example: “if the upper line is twice as long as the lower line, you should respond 2; if the upper line is one-third as long as the lower line, you should respond 1/3” (Parker et al., 1975, p. 197); or “if the second tone seemed ‘one-fourth’ as high as the first, the subject should respond ‘25’; if it seemed ‘half’ as high, ‘50’; ‘twice’ as high, ‘200’; and ‘four times’ as high, ‘400’.” (Elmasian & Birnbaum, 1984, p. 532). The resulting response space was therefore a concatenation of two scales – small ratios where the larger magnitude was the reference stimulus, and big ratios where the smaller magnitude was. A recent paper by Mertens et al. (2021) noted a potential problem with this approach:

We assume that a problem arises because of the asymmetry of the response scale, where for stimuli with a lower intensity compared to the reference, the scale ranges from 0 to 10, but for stimuli with a higher intensity, it ranges from 10 to infinity. Thus, for stimuli that are less intensive than the reference only a limited range is available, while for more intensive stimuli an unlimited range is available. (Mertens et al., 2021, p. 2349)

A possible consequence of this ‘asymmetry’, these authors argued, is a systematic bias in the estimation of the psychophysical function. They reasoned that the comparatively limited range of numbers available for quantifying magnitudes less intense than the reference, alongside a general inability of people to think in terms of ratio notation, could plausibly result in less intense stimuli being assigned relatively more extreme estimates. In order to test for this bias, Mertens et al. (2021) reported brightness and saturation estimation experiments in which (a) the response scale was reversed (so, brighter stimuli were associated with lower numeric estimates), and (b) a ‘unidirectional’ response scale was used (i.e., one incorporating big ratios only). Results from these procedures differed from those obtained using the ‘standard’ magnitude estimation procedure (the concatenated ratio scale), leading the authors to conclude that “the typical power functions that emerge when using a standard magnitude estimation procedure might be biased due to difficulties experienced by participants to think in ratios” (Mertens et al., 2021, p. 2347). For present purposes, their results suggest subjects approached the quantification of big and small ratios differently, rendering the distinction an important one for the study of psychophysical comparison.

Another limitation of prior perceptual comparison studies, addressed by Grace et al. (2018), is that all involved explicit, numeric estimation of differences and ratios – subjects were always instructed which operation to employ, and responded using numbers. Grace et al. noted that while organisms have presumably been comparing perceptual magnitudes for eons, mathematics only emerged in human culture some 5,000 years ago (Neugebauer, 1969). Investigations into the underlying nature of these comparisons, therefore, should ideally avoid explicit use of mathematical concepts. Drawing on behavioural learning methodologies, they devised a non-symbolic, non-verbal task designed instead to engage implicit learning processes. Human subjects viewed pairs of stimuli that varied in brightness, area or numbers of dots, and responded by clicking along an unmarked horizontal response bar. Visual feedback was provided based on the ratio or difference of the nominal stimulus values, linearly mapped along the response bar. In this sense Grace et al.’s paradigm can be conceptualised as a cross-modality matching task (insofar as the differences and ratios of brightnesses, areas and numerosities were mapped to line segments). Their approach thus offered a means to test empirically whether differences or ratios were able to be learned more readily, while avoiding problems stemming from the indeterminacy of the operations in their numeric form (as articulated by Torgerson), and under conditions in which subjects did not use their mathematical knowledge. Contrary to the majority of previous research in this space, their results showed subjects were able to estimate both differences and (big) ratios with similar speed and accuracy, and regression analyses of data at an individual level suggested that in most cases, both operations contributed to responding.

Like de Graaf and Frijters (1988) and Masin et al. (Masin, 2013, 2014; Masin et al., 2019; Masin & Brancaccio, 2017), Grace et al.’s (2018) task employed big ratios only (since the feedback given in the ratio-trained condition always corresponded to the scaled ratio of the larger to the smaller magnitude presented). Thus, the same conceptual limitation described above applies to their task. Additionally, it is possible that their subjects performed accurately by exemplar learning; that is, by associating locations on the response bar with particular stimulus pairs, rather than responding based on the perceived differences and ratios of the magnitudes presented. Grace et al. (2018) tested for exemplar learning in a second experiment with transfer trials, in which novel pairs were presented in the final block without feedback. On average, responses on transfer trials were close to predictions based on the trained values, suggesting that subjects learned differences and ratios rather than particular exemplars. However, a limitation of this transfer design is that it does not provide a strong test at the level of individual subjects. Thus, it is unclear whether subjects might have used different response strategies – that is, some learned differences or ratios while others responded based on generalisation from exemplars. The possibility of heterogeneity across individuals is suggested by research on function learning (DeLosh et al., 1997; McDaniel et al., 2014). Because Grace et al.'s (2018) task is similar to function learning – subjects learned to make a continuous response based on a pair of stimulus values – it is possible that individuals may have used different response strategies.

## The current research

Our study extends the experimental paradigm developed by Grace et al. (2018) in two key ways. First, in light of the foregoing considerations, big and small ratios are treated as distinct operations, with each tested separately (alongside differences) under the same conditions within Grace et al.’s implicit learning procedure. Brightness (Experiment 1a) and line length (Experiment 1b) stimuli were used to test intensive and extensive magnitudes, respectively, with modal comparisons planned. Like Grace et al.'s (2018) initial study, we hypothesised that subjects trained with the comparative operation employed by the perceptual system would learn the task more quickly, perform more accurately, and exhibit exclusive control by that operation under regression analyses of individual responses.

Second, we sought to provide a stronger test of whether subjects were learning quantitative relationships between magnitudes by introducing an artificial discrepancy between the ‘correct’ response locations associated with each operation, and the feedback given. Experiment 2 was identical to Experiment 1, except that random noise was added to the feedback presented. Noise values were sampled once for the exemplar set, so that multiple presentations of the same exemplar had the same feedback value. Following the rationale of classic prototype studies in categorisation (Posner et al., 1967; Posner & Keele, 1968), if subjects were learning to respond based on differences or ratios they should show a stronger tendency to respond based on the underlying values rather than to the noisy feedback: that is, they should effectively ignore or suppress the noise to some degree. By contrast, if subjects were responding on the basis of exemplars, their learning should include the noise. An advantage of this design over the transfer trials reported in Grace et al.’s (2018) initial study is that whether the noise is learned or suppressed can be tested at the level of individual subjects. We hypothesised that subjects would ignore or suppress the noise, and respond based on the underlying difference, big ratio, or small ratios of the brightnesses and line lengths presented.

## Experiments 1a and 1b

In 336 trials subjects compared pairs of circles that varied in brightness (Experiment 1a) or pairs or lines that varied in length (Experiment 1b). Eight different stimulus magnitudes were used in each experiment, with each of the 28 possible (non-identical) pairs thereof presented 12 times in randomised order over four blocks. Subjects compared the stimuli by making a mouse click along a horizontal response bar that contained no markings. They were randomly assigned to one of three groups in which visual feedback on each trial was provided based on the scaled difference, big ratio, or small ratio of the stimulus values. On the basis of this feedback, subjects were instructed to learn to compare the stimuli as accurately as they could.

We used correlations and ANOVAs to compare how quickly and accurately each group learned to compare the magnitudes, and regressions to determine the best model of average responding. On an individual level, multiple regressions were planned to determine which operation(s) most strongly predicted responding, with ‘correct’ values entered alongside those corresponding to the untrained operation(s) as predictors of average responses given for each pair. As in Grace et al. (2018), we hypothesised that if a single comparative operation is computed by the perceptual system, and if the operation in question is a difference, big ratio, or small ratio, then subjects implicitly trained to produce that operation should exhibit better performance on this task, and exclusive control by the trained operation.

### Method

#### Participants

Twenty-eight psychology students participated in Experiment 1a (8 M, 20 F; *M*_*age*_ = 26.1 years), and 40 participated in Experiment 1b (7 M, 33 F, *M*_*age*_ = 23.2 years). None were familiar with the purpose of the research, and they each received course credit or a NZD$15 shopping voucher as incentive. All reported normal or corrected-to-normal vision.

#### Materials

Following Grace et al. (2018) and Rule et al. (1981), in each experiment the chosen stimulus values were equally spaced according to a square-root transformation that enhanced ordinal discrepancies between ratios and differences.

In Experiment 1a the stimuli were pairs of circles with greyscale values of 50, 66, 84, 104, 126, 150, 176 and 205. These were the same as those used by Grace et al. (2018, Experiments 1a and 2a), sampled from an eight-bit monochrome palette with possible values ranging between 0 and 255. The circles were each 6 cm in diameter (6.84 visual angle), and were displayed horizontally against a black background, 8 cm from the top of the screen and separated by 3.3 cm.

In Experiment 1b the stimuli were pairs of yellow (R255, G255, B0) lines 1 mm in width, presented side-by-side against a black background. Their lengths were 22, 29, 45, 58, 75, 94, 115 and 137 mm. To discourage the use of direct measurement strategies (such as visual ‘anchors’ against which the lines could be compared), the lines were presented at randomised angles (which differed by at least 30° within pairs). Their midpoints were positioned 18 cm apart and 10 cm from the top of the screen. Subjects were instructed to ignore the lines’ angles, and respond based on their lengths only.

Located 6.5 cm below the stimulus pairs was a 16.5 cm (length) × 2 mm (width) grey response bar that contained no markings. For each experiment, nominal difference, big ratio, and small ratio values were calculated for each stimulus pair, and linearly mapped along the response bar (0 to 1, from left to right) such that an identical pair would be mapped to 0 (far left), and the most ordinally distant pair (i.e., that comprising the dimmest and brightest circles, or shortest and longest lines in the set) was mapped to 1 (far right). Scaled differences were calculated by dividing the nominal difference of each pair by the difference of the most extreme pair in the set. For Experiment 1a, this was calculated as: Difference_scaled_ =([max]-[min])/(205-50), and for Experiment 1b: Difference_scaled_=([max]-[min])/(350-50). Scaled ratios (big and small) were calculated by dividing each nominal ratio by the ratio of the most extreme pair, after subtracting 1 from the numerator and denominator. For big ratios, this had the effect of ‘zeroing’ the scaled values (so that an identical pair would be mapped to 0). For small ratios, it had the effect of inverting the order of the scaled values to be consistent with differences and big ratios (so that regardless of the operation trained, more similar pairs would be mapped to the left, and less similar pairs toward the right of the response bar). For Experiment 1a, the calculations were Big ratio_scaled_=([max]/[min] - 1)/((205/50) - 1) and Small ratio_scaled_= ([min]/[max] - 1)/((50/205) - 1). For Experiment 1b, they were Big ratio_scaled_=([max]/[min] - 1)/((350/50) - 1) and Small ratio_scaled_=([min]/[max] - 1)/((50/350) - 1). As in Grace et al. (2018), the difference, big ratio or ratio of each pair in the set was therefore represented as a position on the response bar relative to the difference or ratio of the most extreme pair encountered. This approach underscored the distinct metric properties of differences, big ratios, and small ratios as sets, rendering the operations empirically distinguishable in a way that wouldn’t be possible if they were expressed as numbers (due to the indeterminacy noted by Torgerson). Table 1 shows the greyscale (left panels) and pixel length (right panels) values for each stimulus pair, and their raw and scaled differences, big ratios, and small ratios.

**Table 1:** Stimulus values used for Experiments 1 and 2

Experiments 1a and 2a – Brightness								Experiments 1b and 2b – Line length							
Greyscale		Nominal values			Scaled values			Pixel length		Nominal values			Scaled values		
Left	Right	Diff.	Big ratio	Sm. ratio	Diff.	Big ratio	Sm. ratio	Left	Right	Diff.	Big ratio	Sm. ratio	Diff.	Big ratio	Sm. ratio
50	66	16	1.32	0.76	.10	.10	.32	50	76	26	1.52	0.66	.09	.09	.40
50	84	34	1.68	0.60	.22	.22	.54	50	108	58	2.16	0.46	.19	.19	.63
50	104	54	2.08	0.48	.35	.35	.69	50	145	95	2.90	0.34	.32	.32	.76
50	126	76	2.52	0.40	.49	.49	.80	50	188	138	3.76	0.27	.46	.46	.86
50	150	100	3.00	0.33	.65	.65	.88	50	237	187	4.74	0.21	.62	.62	.92
50	176	126	3.52	0.28	.81	.81	.95	50	291	241	5.82	0.17	.80	.80	.97
50	205	155	4.10	0.24	1	1	1	50	350	300	7.00	0.14	1	1	1
66	84	18	1.27	0.79	.12	.09	.28	76	108	32	1.42	0.70	.11	.07	.35
66	104	38	1.58	0.63	.25	.19	.48	76	145	69	1.91	0.52	.23	.15	.56
66	126	60	1.91	0.52	.39	.29	.63	76	188	112	2.47	0.40	.37	.25	.70
66	150	84	2.27	0.44	.54	.41	.74	76	237	161	3.12	0.32	.54	.35	.79
66	176	110	2.67	0.38	.71	.54	.83	76	291	215	3.83	0.26	.72	.47	.86
66	205	139	3.11	0.32	.90	.68	.90	76	350	274	4.61	0.22	.91	.60	.91
84	104	20	1.24	0.81	.13	.08	.25	108	145	37	1.34	0.74	.12	.06	.30
84	126	42	1.50	0.67	.27	.16	.44	108	188	80	1.74	0.57	.27	.12	.50
84	150	66	1.79	0.56	.43	.25	.58	108	237	129	2.19	0.46	.43	.20	.64
84	176	92	2.10	0.48	.59	.35	.69	108	291	183	2.69	0.37	.61	.28	.73
84	205	121	2.44	0.41	.78	.46	.78	108	350	242	3.24	0.31	.81	.37	.81
104	126	22	1.21	0.83	.14	.07	.23	145	188	43	1.30	0.77	.14	.05	.27
104	150	46	1.44	0.69	.30	.14	.41	145	237	92	1.63	0.61	.31	.11	.45
104	176	72	1.69	0.59	.46	.22	.54	145	291	146	2.01	0.50	.49	.17	.59
104	205	101	1.97	0.51	.65	.31	.65	145	350	205	2.41	0.41	.68	.24	.68
126	150	24	1.19	0.84	.15	.06	.21	188	237	49	1.26	0.79	.16	.04	.24
126	176	50	1.40	0.72	.32	.13	.38	188	291	103	1.55	0.65	.34	.09	.41
126	205	79	1.63	0.61	.51	.20	.51	188	350	162	1.86	0.54	.54	.14	.54
150	176	26	1.17	0.85	.17	.06	.20	237	291	54	1.23	0.81	.18	.04	.22
150	205	55	1.37	0.73	.35	.12	.35	237	350	113	1.48	0.68	.38	.08	.38
176	205	29	1.16	0.86	.19	.05	.19	291	350	59	1.20	0.83	.20	.03	.20

The greyscale values of 28 pairs of circles, and the pixel lengths of 28 pairs of lines are shown, alongside their nominal differences and big/small ratios, and their scaled differences and big/small ratios (linearly mapped between 0 and 1)

#### Procedure

Participants were tested in small groups across several sessions. They were randomly assigned to difference, big ratio or small ratio groups (respective *n*s were 10, 10 and 8 for Experiment 1a and 12, 13 and 15 for Experiment 1b).

The following instructions were given: “In this experiment you’ll see pairs of [circles/lines] on the screen, with a horizontal response bar underneath. For each pair, you need to compare the [brightnesses/lengths] of the [circles/lines] by clicking on the horizontal bar. The purpose is to learn to compare the [brightnesses/lengths] as accurately as you can. You’ll receive feedback after each response, showing the correct response location for that pair. Correct responses are followed by green feedback, and incorrect responses by red feedback. If your response is incorrect, you’ll have a chance to repeat with the same pair of [circles/lines]. You should respond at whatever pace feels comfortable and natural for you.” In Experiment 1b they were also instructed “the lines will appear at random angles. The angles aren’t important. You should respond based on the lengths of the lines only”.

**Fig. 2:**
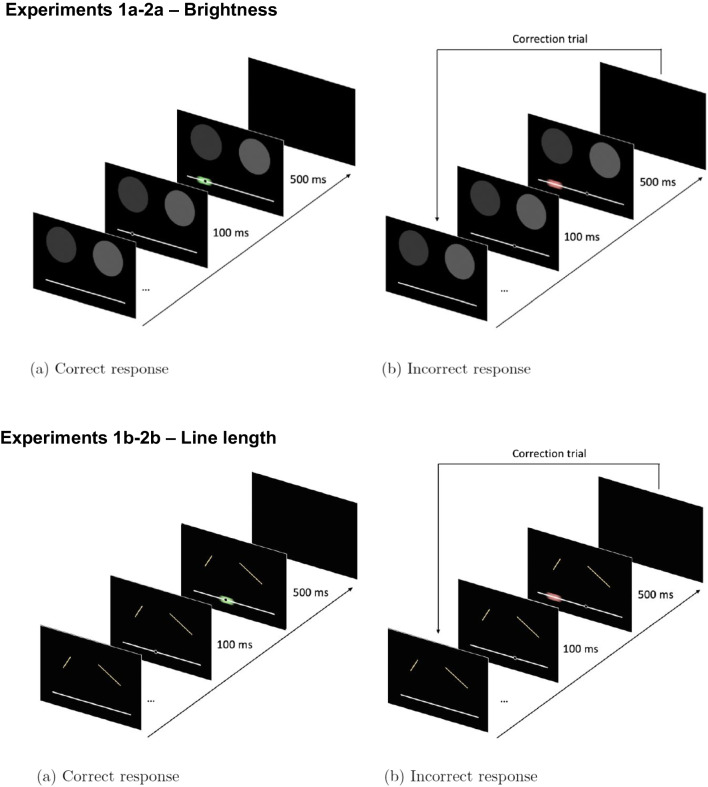
Procedure for each trial in Experiments 1a and 2a (**top panel**) and Experiments 1b and 2b (**bottom panel**). Ovals centred on correct response location followed 100 ms after subject’s response was inputted – green if their response was within 7% of correct location; red if not. Incorrect responses were followed by a single correction trial with the same stimulus pair

There were four blocks of 84 trials each, separated by brief rest periods. Each of the 28 possible (non-identical) stimulus pairs was presented on three trials during each block, giving a total of 336 trials comprising 12 repetitions of each pair. The smaller (i.e. dimmer/shorter) stimulus was always presented on the left, and the order of stimulus pairs was randomised within each block. Using a mouse, participants indicated their response by clicking on the horizontal response bar. There was no time limit to respond. After a 100-ms delay, feedback consisting of an oval (10-mm diameter), centred on the training value (i.e., scaled difference, big ratio or small ratio) and extending 7% of the response bar in either direction, was presented. Where the participant’s response fell within this oval (‘correct’ response), it was green. Where it fell outside 7% of the designated value (‘incorrect’ response), the oval was red. After 500 ms, the stimuli and response bar were removed, and the next trial began after a 2-s interval. Incorrect responses were followed by a single correction trial in which the same stimulus pair was presented. Responses on correction trials were omitted for all analyses. Figure 2 illustrates the procedure used for each trial.

Data from subjects who exhibited unsystematic responding, or failed to meet a minimal level of task engagement, were planned to be excluded. This was pre-determined as an overall correlation with trained values of *r* < .50 over Blocks 2–4 of the experiment. Where large numbers of exclusions occurred, additional sessions were run where required to avoid an unbalanced design.

### Results

Data were omitted from ten subjects in Experiment 1b whose correlations with trained values were *r* < .50, ranging between -.21 and .44. Two were from the difference group, three from the big ratio group, and five from the small ratio group.

In order to preclude the use of direct measurement strategies in this task, the lines in Experiment 1b were each presented at randomised angles (with each pair differing by at least 30°). Although subjects were instructed to respond based only on the lines’ lengths (ignoring the angles), the difference of each pair’s angles was correlated against responses to determine whether the angles were in fact ignored. The correlation was not significant (*r* = .01, *p* = .09), indicating the variation in angles did not impact subjects’ comparisons.

**Fig. 3:**
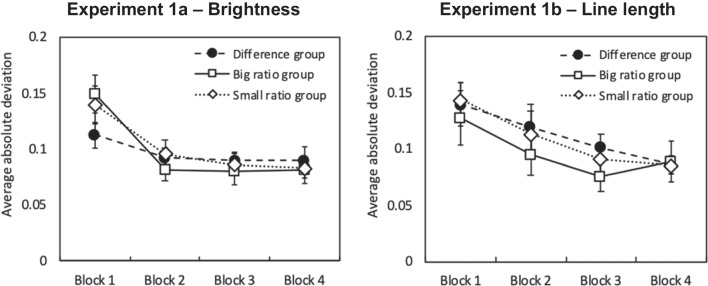
Average absolute deviations from trained values in Experiments 1a and 1b, by block. Bars indicate one standard error

Figure 3 shows the average absolute deviation of responses from trained values by group and block for Experiment 1a (left panel) and 1b (right panel). A repeated-measures ANOVA, with Block as within-subjects factor and Group (difference vs. big ratio vs. small ratio feedback) and Modality (brightness or line length) as between-subjects factors, found a significant main effect of Block (Greenhouse-Geisser adjusted), *F*(1.84, 95.85) = 37.36, *p* < .001, *η*^2^ = 0.42. There were no main effects of Group or Modality (*p*s > .43), nor any significant interactions (*p*s > .21). These results indicate that overall performance in the task improved as the experiment progressed, and that this trend was consistent across groups and modalities. Subsequent analyses pooled responding across Blocks 2–4.

**Fig. 4:**
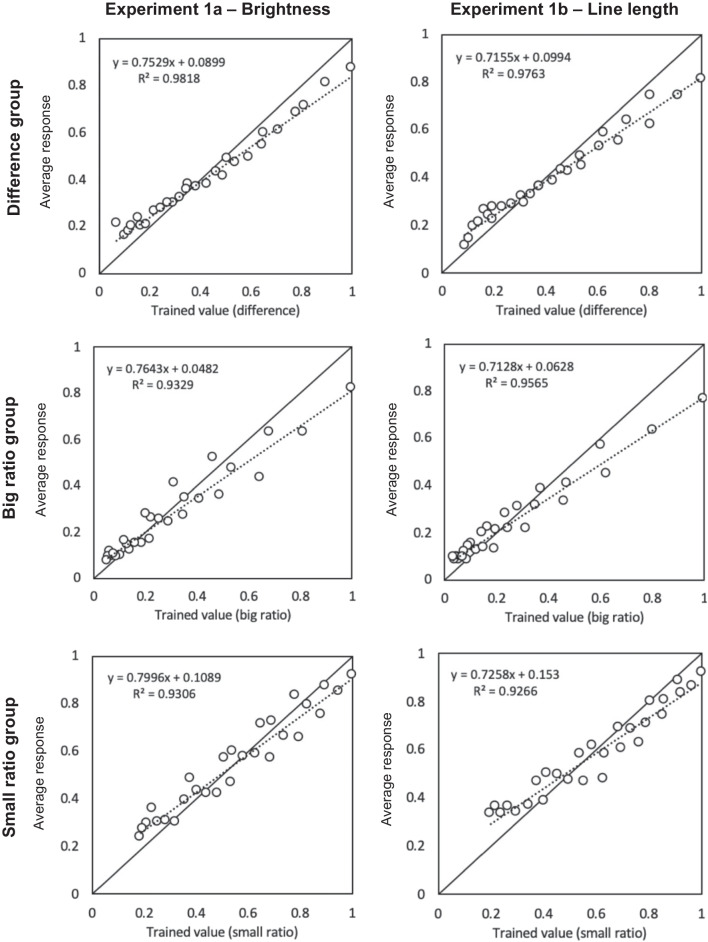
For each brightness and line length pair, trained value (x axis) is plotted against average response over Blocks 2–4 for each group. Solid diagonal lines indicate perfect accuracy; dotted lines show best-fitting regressions (equations and *R*^2^s also shown)

Figure 4 plots the average response for each stimulus pair over Blocks 2–4 (y axes), against the trained values (x axes), for each group (rows) and modality (columns). Average individual correlations with trained values were high (*M* = .92 [95% *CI*: .89, .94]), and did not significantly differ between groups (*p* = .729). Following Chesney and Matthews (2018, 2022), the linear model depicted in Fig. 4 was tested against three alternative models of responding: a logarithmic model (*y* = B × ln(stimulus) + C); a power model (*y* = C × stimulus^B^); and a cyclical power model (*y* = (stimulus^B^ / (stimulus^B^ + (range - stimulus)^B^ )) × range), where range = 100, and best-fitting B and C parameters were obtained using the Solver function in Microsoft Excel. The cyclical power model was initially proposed by Spence (1990) to account for systematic under- and over-estimation of respective lower and upper parts of the range in proportional estimation tasks, and may account for the shallow slopes observed in the regression lines in Fig. 4. Table 2 reports the results of these analyses. The linear model accounted for the most variance in the average responses of all groups. Like Grace et al. (2018), and Morton et al. (2024), who also observed regression slopes < 1 in their average data on similar tasks, we attribute this to a general conservatism in responding: subjects approximated the linear mapping trained, they just tended to avoid the extreme ends of the response scale.

**Table 2:** Regressions of alternative response models on average group responses in Experiments 1a and 1b

	Brightness (Experiment 1a)			Line length (Experiment 1b)		
Group	Difference	Big ratio	Small ratio	Difference	Big ratio	Small ratio
Linear model						
B	0.765	0.764	0.800	0.715	0.713	0.726
C	0.083	0.048	0.109	0.099	0.063	0.153
*R*^2^	**0.987**	**0.933**	**0.931**	**0.976**	**0.957**	0.927
Log model						
B	0.276	0.207	0.376	0.258	0.166	0.353
C	0.699	0.595	0.817	0.678	0.541	0.804
*R*^2^	0.876	0.817	0.883	0.892	0.787	0.844
Power model						
B	0.804	0.831	0.787	0.744	0.772	0.724
C	0.829	0.784	0.893	0.787	0.740	0.865
*R*^2^	0.961	0.933	0.928	0.955	0.949	0.911
Cyclical power model						
B	0.752	0.883	0.746	0.712	0.854	0.646
C	-	-	-	-	-	-
*R*^2^	0.943	0.842	0.921	0.894	0.818	**0.924**

The linear model of responding was compared against three alternative models by regressing each on average response of each group in Experiments 1a and 1b. Logarithmic model: *y* = B × ln(stimulus) + C; Power model: *y* = C × stimulus^B^; Cyclical power model: *y* = (stimulus^B^ / (stimulus^B^ + (range - stimulus)^B^ )) × range. Boldface indicates highest *R*^2^

Following Grace et al. (2018), a series of multiple regressions were conducted to determine whether responding at the individual level was best predicted by one operation or two. For each subject in the difference groups, two multiple regressions were performed: scaled differences were entered alongside big ratios as predictors of average response across Blocks 2–4 in the first regression, and alongside small ratios in the second regression. One regression was performed for each subject in the big and small ratio groups, in which differences were entered alongside the trained ratio operation. The total proportion of variance accounted for (*R*^2^), and unstandardised coefficients for each predictor (*b*) are shown for each regression in Table 3.

**Table 3:** Individual multiple regressions of differences + ratios – Experiments 1a and 1b

Obs.	Experiment 1a – Brightness						Experiment 1b – Line length					
	Diff *b*	Big ratio *b*	*R*^2^	Diff *b*	Small ratio *b*	*R*^2^	Diff *b*	Big ratio *b*	*R*^2^	Diff *b*	Small ratio *b*	*R*^2^
	Difference group						Difference group					
1	0.65^***^	0.15	.93	0.62^***^	0.18	.94	0.58^***^	0.33^***^	.97	0.60^***^	0.31^***^	.96
2	0.75^***^	-0.09	.83	1.15^***^	-0.54^***^	.91	0.59^***^	0.42^***^	.94	0.80^***^	0.17	.90
3	0.90^***^	0.04	.95	0.88^***^	0.07	.96	0.62^***^	-0.33^*^	.55	0.81^***^	-0.56^***^	.74
4	0.13	0.37^**^	.78	0.12	0.38^**^	.78	0.76^***^	0.03	.95	0.73^***^	0.07	.96
5	0.93^***^	-0.19	.92	1.11^***^	-0.40^***^	.95	0.70^***^	0.09	.94	0.75^***^	0.02	.94
6	0.96^***^	-0.25	.90	1.10^***^	-0.41^***^	.93	0.73^***^	0.21^*^	.93	0.77^***^	0.15	.93
7	0.56^***^	0.19	.91	0.76^***^	-0.03	.90	0.76^***^	-0.25^*^	.83	0.74^***^	-0.23^*^	.83
8	0.74^***^	-0.01	.89	0.76^***^	-0.04	.89	0.73^***^	0.14^*^	.97	0.79^***^	0.07	.97
9	1.20^***^	-0.37^***^	.97	1.21^***^	-0.37^***^	.97	0.69^***^	0.00	.95	0.68^***^	0.01	.95
10	0.65^***^	0.23^*^	.95	0.56^***^	0.33^***^	.96	0.25	0.29^*^	.70	0.49^**^	-0.01	.64
	Big ratio group			Small ratio group			Big ratio group			Small ratio group		
1	0.40^***^	0.47^***^	.97	0.43^***^	0.41^***^	.93	0.20^***^	0.73^***^	.98	0.43^***^	0.49^***^	.98
2	0.23^**^	0.79^***^	.98	0.38^**^	0.38^*^	.88	0.33^***^	0.53^***^	.96	0.46^***^	0.22^*^	.90
3	0.19^*^	0.76^***^	.97	0.40^**^	0.51^***^	.93	0.39^***^	0.60^***^	.97	0.32^***^	0.47^***^	.93
4	0.10	0.78^***^	.95	0.30^**^	0.60^***^	.95	0.34^***^	0.52^***^	.95	0.29^***^	0.54^***^	.96
5	0.75^***^	-0.12	.90	0.35^***^	0.45^***^	.94	0.19^**^	0.59^***^	.94	0.50^***^	0.38^***^	.96
6	0.49^***^	0.43^***^	.97	0.41^***^	0.08	.90	0.24^***^	0.61^***^	.96	0.45^***^	0.10	.89
7	0.64^***^	0.10	.93	0.53^***^	0.37^**^	.93	0.07^*^	0.84^***^	.99	0.16^**^	0.67^***^	.96
8	0.34^***^	0.46^***^	.95	0.40^***^	0.55^***^	.97	0.28^***^	0.45^***^	.93	0.30^***^	0.59^***^	.97
9	0.71^***^	-0.15	.89				0.18^**^	0.06	.69	0.38^***^	0.56^***^	.94
10	0.09	0.37^*^	.62				0.13	0.05	.36	0.01	0.28^*^	.50

For each subject (Obs.), unstandardised coefficients and *R*^2^s are shown for regressions of differences + ratios on responding. *p < .05; **p < .01; ***p < .001

Results for Experiment 1a are shown in the left-hand side of Table 3. For the difference-trained group (top-left quadrant), differences were always significant predictors of responding, with mean (unstandardised) coefficients of 0.75 [95% *CI*: 0.57, 0.93] and 0.83 [95% *CI*: 0.62, 1.04], when regressed alongside big and small ratios, respectively. Ratios contributed little to responding for this group, with mean coefficients not significantly differing from zero (*M*s were 0.01 [95% *CI*: -0.14, 0.15] for big ratios and -0.08 [95% *CI*: -0.29, 0.12] for small ratios). A different pattern of results was seen for the ratio-trained groups (bottom-left quadrant), where responding was influenced by both differences and ratios in most cases. The mean coefficient for the trained operation in the big ratio group was 0.39 [95% *CI*: 0.17, 0.61], and for differences was 0.40 [95% *CI*: 0.24, 0.55]. For the small ratio group, the mean coefficient for small ratios was 0.42 [95% *CI*: 0.31, 0.53], and for differences was 0.40 [95% *CI*: 0.35, 0.45].

Results of Experiment 1b, shown in the right-hand side of Table 3, followed a similar pattern. For the difference group (top-right quadrant), difference coefficients were almost always significant. Means were 0.64 [95% *CI*: 0.55, 0.74] when regressed against big ratios, and 0.72 [95% *CI*: 0.65, 0.78] when regressed against small ratios. Ratios again had non-significant overall influence on responding for this group, with mean coefficients of 0.09 [95% *CI*: -0.06, 0.24] and 0.00 [95% *CI*: -0.15, 0.15] for big and small ratios, respectively. By comparison, the majority of subjects in the big and small ratio groups (bottom-right quadrant) again exhibited control by both the trained ratio and differences, with mean coefficients of 0.50 [95% *CI*: 0.34, 0.66] and 0.43 [95% *CI*: 0.32, 0.54] for the respective ratios trained, and 0.23 [95% *CI*: 0.17, 0.30] and 0.33 [95% *CI*: 0.23, 0.42] for untrained differences. Taken together, patterns of responding over both experiments point to exclusive control by differences when differences were trained, and joint control by differences and ratios when ratios (either big or small) were trained, regardless of modality.

Although the correlations between predictor variables were high (*r*s. = .91), Variance Inflation Factors (VIFs) were within generally accepted tolerance limits for the regressions reported (5.75 for differences + big ratios, and 5.99 for differences + small ratios). Monte Carlo analyses performed by Grace et al. (2018) and Chen et al. (2020) on similar datasets suggest the large and negative coefficients obtained in the difference groups were genuine, and not due to multicollinearity.

### Discussion

Results of Experiment 1 showed that subjects trained to judge differences, big ratios and small ratios of brightnesses and line lengths learned the task with comparable speed, and approximated the target comparative operation with similar levels of accuracy. No disparity was found in terms of task performance according to either the operation trained, or the modality compared. If there is a fundamental comparison operation, these findings suggest that subjects can nonetheless learn the other operations with facility, regardless of whether the magnitudes under comparison are intensive or extensive.

Individual regression results were less uniform, and suggested a possible bias towards difference-based responding overall. The majority of subjects trained with difference feedback showed exclusive control by that operation (13/20), while subjects trained with big or small ratios showed control by both differences and the trained ratio operation in most cases (28/38). These results suggest two influences on responding in the task: the feedback provided, and an underlying predisposition toward difference-based judgements. For subjects in the difference groups, it is possible that each of these influences contributed to strong difference-based responding, with little to no influence of ratios. For the ratio-trained groups, the combination of these factors ostensibly resulted in responding jointly influenced by both ratios and differences (due to the first and second factors, respectively). These patterns of responding were again consistent across modalities, suggesting a common perceptual mechanism for comparing intensive and extensive dimensions.

## Experiments 2a and 2b

In Experiment 1, subjects compared the brightness or length of 28 unique stimulus pairs presented 12 times each over the course of the session. Since each pair was seen more than once, it is possible that rather than learning to respond based on the comparative operation trained, subjects instead adopted an exemplar learning strategy; that is, learned to associate the correct response location with each unique pair. To test for this possibility, random noise was added to the feedback associated with each of the brightness (2a) and line length (2b) pairs. The setup and procedure were otherwise identical to Experiment 1.

Multiple regressions were planned to test whether the subjects ignored or suppressed the added noise. Responses of individual subjects across the 28 pairs were entered into multiple regressions with the trained values (i.e., difference or ratio plus noise) and the added noise component as separate predictors. If subjects responded by exemplar learning, then regression coefficients for the added noise should not differ systematically from zero. However, if subjects were learning the underlying difference or ratio relation, then the noise component should yield significantly negative coefficients, suggesting that subjects were suppressing the noise. In the event the noise was suppressed, we also planned regression analyses identical to those undertaken in Experiment 1, to determine which operation(s) best characterised responding to an individual level, and to test whether this differed across groups and modalities.

### Method

#### Participants

Twenty-five undergraduate psychology students served as subjects in Experiment 2a (8 M, 15 F; *M*_*age*_ = 21.3 years), and 32 in Experiment 2b (10 M, 22 F; *M*_*age*_ = 22.1 years). All reported normal or corrected-to-normal vision. None were familiar with the purpose of the research, and they received course credit or a NZD$15 shopping voucher in exchange for participation.

#### Materials

The stimuli were the same 28 pairs of circles and lines used in Experiments 1a and 1b. Gaussian noise was generated by sampling from a normal distribution *N*(0, .10 x *σ *_*d, b, s*_^2^), where *σ *_*d*_^2^, *σ *_*b*_^2^ and *σ *_*s*_^2^ are the variances of the scaled difference, big ratio, and small ratio values. This noise component was then added to each of the scaled difference, big ratio, and small ratio values for each pair shown in Table 1 (with any values < 0 or > 1 changed to 0 and 1, respectively). The resulting values were used as feedback throughout each experiment, such that the feedback provided deviated from the location associated with the true difference, big ratio or small ratio by up to 10%. The noise distributions were sampled once and the resulting training values were the same across all subjects and presentations of the same pair.

#### Procedure

The procedure was identical to Experiment 1. Subjects were randomly assigned to difference, big ratio or small ratio groups (*n*s were 8, 8 and 9, respectively, for Experiment 2a, and 9, 12 and 11 for Experiment 2b), and were tested in small groups. They were each seated in a cubicle with an HP Elitedesk core i7 computer with a Samsung 22-in. LCD monitor. The monitor had a resolution of 1,680 × 1,050 pixels and a maximum brightness of 300 cd/m^2^ and contrast ratio of 1000:1. The instructions were identical to those given in Experiments 1a and 1b, as were the number and structure of trials completed, as depicted in Fig. 2. Data from correction trials were again omitted from all analyses, and the same exclusion criteria as in Experiment 1 were applied.

### Results

Data were omitted from six subjects on the basis that their correlations with trained and scaled values were less than .50 (*r*s < .49) – two from Experiment 2a and four from Experiment 2b. Two were from the difference groups, two from the big ratio groups, and two from the small ratio groups.

Hierarchical regressions were first conducted to test whether the added noise accounted for significant variance in individual subjects’ responding beyond the trained values. Specifically, for each subject we conducted two regressions in which the trained value was entered at the first step, and the deviation between the scaled difference or ratio and the corresponding trained value (i.e. the noise component) was entered at the second step. Results are shown in Table 4. Noise coefficients were negative for all subjects, and significantly so in 41/51 cases, with a mean of -0.59 [95% *CI*: -0.67, -0.51]. A two-way ANOVA showed no significant difference in coefficients by Group or Modality (*p*s > .10). Since noise coefficients were negative in all cases, significant in the majority, significantly less than zero on average, and uniform across groups and modalities, these results are consistent with the prediction that subjects would generally suppress the noise and respond based on the underlying operation.

**Table 4:** Individual hierarchical multiple regressions of trained values + noise, Experiments 2a and 2b

Obs.	Experiment 2a – Brightness					Experiment 2b – Line length				
	Train *b*	Noise *b*	*R*^2^	*R*^2^_inc._	*p*	Train *b*	Noise *b*	*R*^2^	*R*^2^_inc._	*p*
	Difference group					Difference group				
1	0.82	-0.71	.96	.06	<.001^***^	0.71	-0.61	.97	.06	<.001^***^
2	0.76	-0.76	.96	.08	<.001^***^	0.85	-0.86	.94	.08	<.001^***^
3	0.67	-0.67	.78	.07	.011^*^	0.52	-0.21	.63	.01	.458
4	0.88	-0.45	.85	.02	.066	0.27	-0.24	.56	.03	.176
5	0.63	-0.39	.87	.03	.021^*^	0.70	-0.66	.93	.07	<.001^***^
6	0.57	-0.19	.78	.01	.338	0.57	-0.61	.74	.07	.019^*^
7	0.80	-0.45	.94	.03	.002^**^	0.82	-0.82	.94	.08	<.001^***^
8						0.16	-0.03	.26	.00	.892
	Big ratio group					Big ratio group				
1	0.37	-0.40	.80	.09	.003^**^	0.64	-0.54	.86	.04	.017^*^
2	0.78	-0.94	.91	.13	<.001^***^	0.28	-0.19	.46	.01	.448
3	0.75	-1.00	.91	.15	<.001^***^	0.82	-0.77	.96	.05	<.001^***^
4	0.81	-0.49	.83	.03	.034^*^	0.72	-0.59	.87	.04	.017^*^
5	0.82	-0.52	.88	.04	.009^**^	0.71	-0.76	.86	.06	.004^**^
6	0.81	-0.86	.85	.10	.001^**^	0.88	-0.89	.93	.06	<.001^***^
7	0.81	-0.60	.94	.05	<.001^***^	0.69	-0.76	.89	.07	.001^**^
8						0.79	-0.63	.97	.04	<.001^***^
9						0.85	-0.81	.92	.05	.001^**^
10						0.49	-0.41	.71	.03	.121
11						0.84	-0.77	.92	.05	<.001^***^
	Small ratio group					Small ratio group				
1	0.40	-0.53	.70	.14	.003^**^	0.96	-1.14	.94	.10	<.001^***^
2	0.69	-0.68	.83	.09	.001^***^	0.32	-0.04	.58	.03	.858
3	0.36	-0.38	.37	.05	.190	0.59	-0.63	.79	.07	.010^*^
4	0.68	-1.06	.85	.22	<.001^***^	0.93	-0.88	.98	.07	<.001^***^
5	0.84	-1.03	.93	.15	<.001^***^	0.53	-0.67	.73	.09	.009^*^
6	0.32	-0.43	.62	.12	.010^*^	0.63	-1.00	.67	.12	.006^*^
7	0.39	-0.43	.41	.06	.130	0.66	-0.89	.81	.11	.001^*^
8	0.65	-0.58	.90	.80	< 001^***^	0.97	-1.06	.95	.08	<.001^***^
9	0.70	-0.34	.76	.11	.003^**^	0.76	-0.94	.82	.09	.001^*^

For each subject (Obs.), unstandardised coefficients and *R*^2^s are shown for regressions of differences + ratios on responding. *p < .05; **p < .01; ***p < .001

Having established that subjects were not adopting an exemplar learning strategy, multiple regressions were next undertaken to establish which operation(s) most strongly influenced responding at an individual level, identical to those described in Experiment 1. Results are shown in Table 5.

**Table 5:** Individual multiple regressions of differences + ratios – Experiments 2a and 2b

Obs.	Experiment 2a – Brightness						Experiment 2b – Line length					
	Diff *b*	Big ratio *b*	*R*^2^	Diff *b*	Small ratio *b*	*R*^2^	Diff *b*	Big ratio *b*	*R*^2^	Diff *b*	Small ratio *b*	*R*^2^
	Difference group						Difference group					
1	0.66^***^	0.17^*^	.96	0.80^***^	0.00	.96	0.66^***^	0.07	.97	0.71^***^	0.00	.97
2	0.80^***^	-0.05	.96	0.64^***^	0.13	.96	0.69^***^	0.20^*^	.95	0.70^***^	0.19^*^	.95
3	0.93^***^	-0.29	.80	0.91^***^	-0.27	.80	0.65^***^	-0.18	.63	0.87^***^	-0.45^**^	.74
4	0.94^***^	-0.13	.84	0.71^***^	0.13	.84	0.35^***^	-0.10	.57	0.33^**^	-0.07	.57
5	0.58^***^	-0.02	.81	0.56^***^	0.05	.86	0.85^***^	-0.19^**^	.95	0.88^***^	-0.22^**^	.95
6	0.48^***^	0.05	.75	0.55^***^	-0.03	.75	0.76^***^	-0.23	.77	0.88^***^	-0.39^**^	.82
7	0.78^***^	-0.03	.93	0.87^***^	-0.13	.93	0.65^***^	0.22^**^	.95	0.63^***^	0.25^**^	.96
8	0.66^***^	0.17	.96	0.80^***^	0.00	.96	0.46^***^	-0.39^***^	.67	0.46^***^	-0.39^***^	.68
	Big ratio group			Small ratio group			Big ratio group			Small ratio group		
1	0.25^**^	0.13	.81	0.33^**^	0.09	.77	0.20^*^	0.46^***^	.89	0.20^*^	0.78^***^	.95
2	0.47^***^	0.35^**^	.94	-0.17	0.85^***^	.84	0.34^***^	-0.03	.67	-0.19	0.48^***^	.60
3	0.43^***^	0.36^**^	.94	0.81^***^	-0.41^*^	.71	0.16^**^	0.68^***^	.97	0.41^***^	0.23^**^	.91
4	0.20	0.62^***^	.92	0.45^***^	0.26^*^	.89	0.46^***^	0.31^***^	.98	0.09^*^	0.85^***^	.99
5	0.25^*^	0.57^***^	.95	0.00	0.84^***^	.92	0.27^**^	0.47^***^	.90	0.41^***^	0.17	.87
6	0.30^*^	0.55^***^	.92	0.42^***^	-0.08	.79	0.31^***^	0.60^***^	.97	0.66^***^	0.05	.88
7	0.60^***^	0.21^*^	.94	0.86^***^	-0.43^**^	.78	0.34^***^	0.38^***^	.96	0.22^*^	0.47^***^	.84
8				-0.07	0.71^***^	.90	0.09	0.71^***^	.97	0.16^*^	0.83^***^	.95
9				0.76^***^	-0.02	.91	0.21^*^	0.66^***^	.94	0.47^***^	0.35^***^	.92
10							0.51^***^	0.03	.96			
11							0.32^***^	0.55^***^	.97			

For each subject (Obs.), unstandardised coefficients and *R*^2^s are shown for regressions of differences + ratios on responding. *p < .05; **p < .01; ***p < .001

The top-left quadrant of Table 5 shows the results of two regressions performed for each subject in the difference group for brightness. Like Experiments 1a and 1b, differences dominated responding for this group. Their coefficients were significant predictors of responding for all subjects, with means of 0.74 [95% *CI*: 0.61, 0.87] when regressed against big ratios, and 0.72 [95% *CI*: 0.61, 0.83] when regressed against small ratios. Neither big nor small ratios had significant influence on responding overall, with mean coefficients of -0.04 [95% *CI*: -0.15, 0.06] and -0.02 [95% *CI*: -0.12, 0.09], respectively. Responding in the ratio-trained groups (bottom-left quadrant) was more heterogenous. The big ratio group followed the trend observed in Experiment 1, exhibiting medium-sized coefficients for both big ratios and differences in most cases (*M*s = 0.40, [95% *CI*: 0.26, 0.54] and 0.36 [95% *CI*: 0.25, 0.47], respectively). Subjects in the small ratio group, on the other hand, tended to respond based on small ratios (as seen in cases 2, 5 and 8), or differences (cases 1, 3, 6, 7 and 9), but not both. Mean coefficients were 0.20 [95% *CI*: -0.13, 0.53] for small ratios and 0.38 [95% *CI:* 0.12, 0.63] for differences (with the large confidence intervals here reflecting the noted variability in responding).

Experiment 2b (right-hand side of Table 5) was more consistent with previous results. Subjects trained with differences (top-right quadrant) mostly exhibited exclusive control by that operation, with mean coefficients of 0.63 [95% *CI*: 0.52, 0.74] and 0.68 [95% *CI*: 0.54, 0.82] when regressed alongside big and small ratios, respectively. Big and small ratios yielded mean coefficients that did not significantly differ from zero (-0.08 [95% *CI*: -0.23, 0.08] and -0.14 [95% *CI*: -0.32, 0.05]). In the ratio groups (bottom-right quadrant), responding was again jointly controlled by both the trained ratio and differences in most cases, with mean coefficients of 0.44 [95% *CI*: 0.29, 0.59] and 0.29 [95% *CI*: 0.22, 0.37] for the big ratio group, and 0.42 [95% *CI*: 0.23, 0.61] and 0.34 [95% *CI*: 0.18, 0.51] for the small ratio group. Overall, these response patterns are similar to those seen in Experiment 1, despite the noisy feedback. Responding tended to be based solely on differences when differences were trained, and (with the exception of subjects trained with noisy small ratios of brightnesses), on both differences and ratios when ratios were trained.

A four-way, repeated-measures ANOVA combining the individual regression results obtained in Experiments 1 and 2 was conducted to formally test the effect of feedback and modality on the extent to which differences and ratios influenced responding overall. Operation (regression coefficients for the trained and untrained operations^1^) was entered as a between-subjects measure, and Group (difference-, big ratio-, or small ratio-trained), Noise (accurate or noisy feedback) and Modality (brightness or line length), as between-subjects measures. The dependent variable was the magnitude of the regression coefficient in each case (i.e. the relative extent to which each operation influenced individual responding). These are the values shown in Tables 3 and 5. If the operation trained had an overall impact on the relative influence of differences and ratios on responding, a significant interaction should be found between Operation and Group. Likewise for Modality and Noise. Three- and four-way interactions between the predictor variables were also tested.

There was a significant main effect of Operation, *F*(1, 97) = 43.93, *p* < .001, *η*^2^ = 0.31, and a significant interaction between Operation and Group, *F*(2, 97) = 23.42, *p* < .001, *η*^2^ = 0.33, indicating that the trained operation dominated responding more for the difference group than the ratio groups (*M*s_trained_ were 0.69 [95% *CI*: 0.60, 0.78], 0.43 [95% *CI*: 0.34, 0.52], and 0.38 [95% *CI*: 0.29, 0.47] for the difference, big ratio, and small ratio groups, respectively, while *M*s_untrained_ were 0.00 [95% *CI*: -0.08, 0.07], 0.32 [95% *CI*: 0.25, 0.39], and 0.34 [95% *CI*: 0.27, 0.41], where the ‘untrained’ operation for the difference group was big ratios). No other interaction terms were significant (*p*s > .18), indicating this result was consistent across modalities and unaffected by addition of random noise to the feedback. The same analysis with small ratios entered as the untrained operation for the difference group showed similar results: the main effect of Operation was significant, *F*(1, 97) = 49.58, *p* < .001, *η*^2^ = 0.34, as was the interaction between Operation and Group, *F*(2, 97) = 28.90, *p* < .001, *η*^2^ = 0.37. The other interaction terms were not (*p*s > .18). Here the mean coefficient for trained values for the difference group was 0.74 [95% *CI*: 0.64, 0.83], and for the untrained operation (in this case small ratios) was -0.06 [95% *CI*: -0.14, 0.02]. A subsequent ANOVA omitting the difference groups showed no main effect of Operation (*p* = .19), nor any significant interactions (*p*s > .06), indicating that for both the big and small ratio groups, there was no significant difference in terms of the relative influence of differences and (trained) ratios on responding, for either modality or feedback type.

### Discussion

The main objective of Experiment 2 was to determine whether responding in the task was based on the operation trained, or resulted from exemplar learning. This was achieved by adding 10% random noise to the feedback used for each group. Hierarchical regression analyses of individual responses showed coefficients for the noise component were always negative and statistically significant in the majority of cases (41/51), indicating subjects ignored or suppressed the noise and responded according to the perceived difference (and)/or ratio of the stimulus values presented, thus ruling out exemplar learning.

Regressions of individual responses against scaled differences and ratios produced results largely consistent with Experiment 1. For the majority of subjects trained with noisy difference feedback (12/15), responding was controlled by differences only. The majority of subjects trained with noisy ratios, on the other hand, showed joint control by both ratios and differences (20/36), although subjects trained with noisy small ratios of brightnesses tended to respond based on small ratios only (3/9) or differences only (5/9). This finding suggests that the small ratio operation may have been more difficult to learn when feedback for brightnesses in particular was noisy, leading some subjects to adopt a difference-based response strategy. A tendency towards a default difference-based strategy is supported by ANOVAs on regression coefficients across both experiments, which found the influence of the trained operation was significantly greater than that of the untrained operation(s) for the difference groups, but not for the ratio groups, suggesting, as in Experiment 1, an overall predisposition toward difference judgements. No significant overall effects of modality or noise were found, however (nor any significant interactions), indicating consistency across intensive and extensive judgements, and no discernible impact of the added noise on response patterns in general.

## General discussion

Depending on the order two positive magnitudes are operated on, their ratio can take one of two possible values: a ‘small ratio’ ranging between 0 and 1, or a ‘big ratio’ ranging between 1 and infinity. Because the latter is unbounded above while the former is not, these respective scales have different metric properties that are of consequence for ratio estimation studies, which typically involve analysis of ratio judgements across sets of stimulus pairs, rather than single exemplars. The current research represents the first psychophysical treatment of big and small ratios as separate comparative operations. The implicit learning paradigm developed by Grace et al. (2018) provided a robust means to determine which (if either) ratio type characterises comparative judgements. Because it did not involve explicit instruction or the use of numbers, this approach had the advantage of eliciting difference, big ratio, or small ratio judgements from subjects without their relying on any antecedent mathematical knowledge. Potential issues arising from difficulty understanding and articulating ratios, as suggested by Mertens et al. (2021), or ambiguity arising from free transformation of the instructed operation, noted by Torgerson (1961) were thus avoided.

Results from Experiment 1, which was a basic replication of Grace et al. (2018) with the addition of a small ratio-trained group, showed that subjects learned to accurately approximate differences, big ratios and small ratios of brightnesses and line lengths with similar speed and accuracy. If the perceptual system does confer a preference for one ratio type over the other (or indeed over differences), it was not able to be determined by aggregate metrics of task performance. Individual regression results, however, suggested a possible bias overall towards difference judgements. The majority of subjects trained with difference feedback showed exclusive control by differences, while the majority trained with big or small ratios showed control by both (trained) ratios and differences, with the latter influencing responding in these groups to similar extents. The same analyses from Experiment 2 showed broadly consistent results. In contrast, Grace et al. (2018) reported evidence of control by both operations for the majority of subjects in *both* difference and (big) ratio conditions in their experiments. A bias towards differences is largely consistent with the majority of prior studies of perceptual comparison, though an ability to learn and accurately approximate ratio judgements of both types was evident, indicating a clear capacity for the evaluation of all three comparative operations.

 Masin et al. (2019) have recently argued that ratio judgements are possible with respect to extensive stimuli only. They propose that this is achieved not by direct estimation, but by a process of mentally counting how many times one length (for example) ‘fits within’ another. Because such a strategy would be unavailable for stimuli that lack extension in space or time, ratio estimation of intensive modalities is problematic. The current results provide evidence against this hypothesis. Subjects in our study quickly learned to judge both big and small ratios of brightnesses (an intensive dimension) without instruction, with a high level of accuracy that was similar to that achieved by their difference-trained counterparts. Analysis of regression coefficients in Experiments 1 and 2 showed no interaction between Modality and Group or Operation, indicating the reported bias toward difference-based comparisons was consistent across both intensive and extensive stimuli. The tendency for subjects trained with noisy small ratios of brightnesses to respond based on either small ratios or differences in Experiment 2a may suggest an increased inclination towards difference-based judgements when comparing intensive stimuli in particular (perhaps induced by the higher level of uncertainty associated with the noisy feedback task), but the capacity for accurate ratio estimation of intensive magnitudes within this paradigm is clearly evident.

The discrepancy between our results and those of Masin et al. (2019) suggests that although subjects can perceive ratios of intensive magnitudes, they struggle when required to use mathematical concepts to describe them. Rational numbers (i.e., fractions) are often a challenging topic for mathematics learners (Lortie-Forgues et al., 2015) and it is possible that difficulties with using numerical ratios might have affected subjects’ responding in Masin et al.’s study. There is evidence that mathematical knowledge is related to performance on ratio tasks with extensive stimuli: Matthews et al. (2016) found that individual differences in judging non-symbolic ratios of numerosities and line lengths were positively correlated with symbolic math competence. An interesting question is whether performance with brightness ratios in our task would be impaired by providing subjects with explicit instructions and numeric feedback. Further research involving symbolic estimation of small ratios in particular would also be instructive. The current results show that subjects can accurately estimate small ratios non-symbolically, but as noted, the capacity for numeric small ratio estimation of perceptual magnitudes is relatively unexplored.

The secondary hypothesis addressed in the current research was that subjects in Grace et al.’s (2018) task learned to compare stimuli according to their differences or ratios, and not simply to associate specific stimulus pairs with their correct responses. To compare these alternatives, in Experiment 2 we studied a version of the task in which 10% random noise was added to the feedback provided for each group. The noise was sampled once for the set of stimulus pairs so training values were the same for each presentation of a pair, so that exemplar learning would be evidenced by responses that more closely tracked the noisy feedback than the underlying differences or ratios. We performed hierarchical regressions of individual responses in which the trained values (difference or ratio plus noise) were entered at the first step, and the deviation between the difference or ratio values and the trained value (i.e. the noise component) was entered at the second step. Significant negative coefficients in the majority of cases (and negative coefficients in all) showed that subjects learned to respond based on the underlying differences or ratios, and effectively filtered or suppressed the noisy feedback. This is contrary to the exemplar learning hypothesis and provides strong evidence that relational learning occurred. A potential downside of the effectiveness with which subjects were able to learn to compare magnitudes using feedback in our task is that this may have obscured any underlying tendency toward difference- and/or ratio-based judgements on the part of the perceptual system. To this end, it would be instructive to test whether differences and/or ratios are able to be approximated under similar conditions without feedback to guide responding.

Overall, our findings show that subjects can systematically compare intensive and extensive magnitudes based on differences, big ratios or small ratios, without engaging in any explicit mathematical reasoning. This suggests that the perceptual system is highly flexible in its capacity for implicit computation on a wide range of represented magnitudes. Recent research by Morton et al. (2024), using the same implicit learning paradigm, showed that subjects could be similarly trained to add brightnesses and line lengths in accordance with the properties of an algebraic group. The ease with which subjects can be trained to produce differences, big ratios, small ratios, and sums of continuous magnitudes, without recourse to mathematical symbolism or procedures, points to a perceptual system with an enhanced capacity for computation of environmental variables. It not only represents perceived magnitudes, but relations between those magnitudes also.

The capacity for implicit computation is likely to be evolutionarily ancient. Many non-human species, including insects, show adaptive behaviour that appears to require the equivalent of arithmetic or algebraic calculation with internally-represented variables (Gallistel, 1990, 2017; Grace et al., 2020). Perhaps the best example is spatial navigation by path integration, which allows species from insects to mammals to keep track of their location relative to a homebase while foraging, using cues related to self-directed motion like solar angle and optic flow (Etienne & Jeffery, 2004; Srinivasan, 2015). The neural circuitry supporting path integration is an active area of research in insect neuroscience (e.g., Green et al., 2017, 2019; Lyu et al., 2022). Other examples of such adaptive behaviour include timing, reinforcement learning, and cue integration (see Grace et al., 2020, for review). Although magnitude comparison in humans may seem far removed from the neural basis of navigation in *Drosophila*, there is a common need to understand the role of computation in perception.

Typically, this role has been regarded as an action or function by a biological system that can be described by a computational model, such as an artificial neural network (e.g., Stone et al., 2017). However, computation may be more deeply connected to perception. Recently, Grice et al. (2024) have argued that arithmetic has a biological basis in perception. Using mathematical proof, they showed that four qualitative conditions – monotonicity, convexity, continuity and isomorphism (MCCI) – were sufficient to identify addition and multiplication over the real numbers uniquely among all possible sets and operations. Grice et al. argued that these conditions were principles of perceptual organisation that are shared with non-humans. The upshot of their proof is that numbers and arithmetic are a formal expression of these conditions, and therefore a natural consequence of how our perception is structured. If the perceptual system ‘contains within it’ the precursors of arithmetic, as Grice et al.’s (2024) account suggests, then its capacity to compute operations such as differences, big ratios, small ratios and sums on a wide range of perceptual stimuli without explicit mathematical framing would not be surprising.

## Data Availability

Data are available (alongside a pre-print of this article) at https://osf.io/preprints/psyarxiv/jhnfu/

## Footnotes

1 Two ANOVAs were conducted; in the first big ratios were entered as the ‘untrained’ operation for the difference group, and in the second small ratios were. The untrained operation for both ratio groups was always differences.
